# SARC-F score compared with hand grip strength for screening possible sarcopaenia among community-dwelling older adults: A multi-centre cross-sectional study in Malaysia

**DOI:** 10.51866/oa.1109

**Published:** 2026-06-26

**Authors:** Chai Li Tay, Chai Chen Ng, Chee Cheong Kee, Nor Faizah Ghazali, Zaleha Jusoh, Thenmoli Palaniyappan, Sie Zin Kong, Boon Kiew Lee, Lorna Kin Tze Chin, Ai Lee Ding, Khalilati Barizah Md Salimun, Srinevasarao Ramanaidu

**Affiliations:** 1 Klinik Kesihatan Simpang, Jalan Kuala Kangsar, Simpang, Taiping, Perak, Malaysia.; 2 Hospital Sultan Ismail, Jalan Mutiara Emas Utama, Taman Mount Austin, Johor Bahru, Malaysia.; 3 National Institutes of Health, Jalan Setia Murni U13/52, Seksyen U13 Setia Alam, Shah Alam, Selangor, Malaysia.; 4 Faculty of Medicine and Health Sciences, Universiti Sains Islam Malaysia, Persiaran Ilmu, Putra Nilai, Nilai, Negeri Sembilan, Malaysia.; 5 Klinik Kesihatan Batu Rakit, Kuala Nerus, Terengganu, Malaysia.; 6 Klinik Kesihatan Kuala Kangsar, Kampung Basong, Kuala Kangsar, Perak, Malaysia.; 7 Klinik Kesihatan Sarikei, Sarikei, Sarawak, Malaysia.; 8 Klinik Kesihatan Tudan, Jalan Tudan Permyjaya, Miri, Sarawak, Malaysia.; 9 Klinik Kesihatan Batu Niah, Batu Niah, Sarawak, Malaysia.; 10 Klinik Kesihatan Senawang, Persiaran Senawang 2, Senawang, Negeri Sembilan, Malaysia.; 11 Klinik Kesihatan Segamat, Jalan Muar, Segamat, Johor, Malaysia.; 12 Klinik Kesihatan Labis, Labis, Segamat, Johor, Malaysia.

**Keywords:** Sarcopaenia, SARC-F, Hand grip strength, Health clinics, Older adults

## Abstract

**Introduction:**

Sarcopaenia is underdiagnosed in primary care due to limitations in available tools. The SARC-F questionnaire is a recommended screening tool, but its diagnostic accuracy among Malaysian community-dwelling older adults is not well established. This study aimed to determine the diagnostic accuracy of the SARC-F score against hand grip strength (HGS) for identifying possible sarcopaenia among community-dwelling older adults.

**Methods:**

A cross-sectional study was conducted from January to November 2024 across 11 public health clinics. Community-dwelling older adults aged ≥60 years were recruited. Sarcopaenia risk was assessed using the SARC-F questionnaire, with scores of ≥4 indicating a high risk. HGS was measured using a Jamar dynamometer, with possible sarcopaenia defined according to the Asian Working Group for Sarcopenia 2019 criteria (<28 kg for men, <18 kg for women). Statistical analysis included the Mann–Whitney U test to compare mean rank distributions of HGS between SARC-F risk groups and Cohen’s kappa to assess the agreement between the SARC-F score- and HGS-based classifications. Receiver operating characteristic (ROC) curve analysis was performed to evaluate the discriminatory performance of the SARC-F score in identifying possible sarcopaenia, using HGS as the reference standard.

**Results:**

Among 578 participants, the median HGS was significantly lower in the high-risk SARC-F group for both men (19.0 kg vs 28.0 kg, P<0.001) and women (14.0 kg vs 18.0 kg, P<0.001). The agreement between the SARC-F score- and HGS-based classifications was poor to fair (kappa=0.219). The SARC-F score demonstrated low sensitivity (34.7%) but high specificity (87.5%); it showed poor discriminative ability in detecting possible sarcopaenia (area under the ROC curve=0.67; 95% confidence interval=0.622–0.710; P<0.001). The positive predictive value was 74.6%; negative predictive value, 55.9%; and overall accuracy, 60.4%.

**Conclusion:**

While a high SARC-F score is significantly associated with low HGS, the low sensitivity of the SARC-F score limits its use as a standalone screening tool in primary care. A two-step approach, using the SARC-F questionnaire followed by objective HGS measurement, may enhance early detection efforts in primary care.

## Introduction

Sarcopaenia, a progressive and generalised skeletal muscle disorder involving accelerated loss of muscle mass and function, is an important contributor to frailty, disability and loss of independence in older adults.^1^ It is associated with adverse outcomes including increased risks of falls, fractures, hospitalisation and mortality, imposing a significant burden on healthcare systems and caregivers.^2^ The prevalence of sarcopaenia among community-dwelling older adults in Asia is substantial, with studies reporting rates ranging from 10% to 27%, highlighting it as a critical public health concern.^3^

Possible sarcopaenia is defined primarily by low muscle strength or physical performance. It affects both underweight and obese older adults. Early detection of possible sarcopaenia is a pivotal first step in the management algorithm proposed by the Asian Working Group for Sarcopenia (AWGS) 2019.^4^ Detailed muscle mass assessment via bioelectrical impedance analysis or dual-energy X-ray absorptiometry is needed to confirm the diagnosis of sarcopaenia.4 Although low hand grip strength (HGS) alone does not confirm sarcopaenia, it allows the identification of individuals with possible sarcopaenia who may benefit from early interventions. Resistance exercise, protein supplementation and fall prevention can be implemented to mitigate progression and improve outcomes.^5^

In the primary care setting, where most older adults seek initial healthcare, a simple and effective screening tool is essential for case finding. According to the AWGS 2019 Consensus and AWGS 2025 Consensus Update, measuring the calf circumference or using the SARC-F questionnaire is recommended as a case-finding approach; individuals with positive findings should subsequently undergo further assessment through HGS measurement or the five-times chair stand test to identify possible sarcopaenia.^4,6^

HGS measurement using a Jamar dynamometer, with possible sarcopaenia defined according to the AWGS 2019 criteria (<28 kg for men, <18 kg for women), is a reliable, objective and non-invasive proxy for evaluating overall muscle strength and is the cornerstone of the AWGS 2019 operational definition for possible sarcopaenia.4 However, routine assessment in busy clinics can be constrained by time, the need for calibrated equipment and trained personnel.

The SARC-F questionnaire is a rapid, self-reported five-item tool assessing strength, assistance in walking, rising from a chair, climbing stairs and falls.^7^ With a score of >4 indicating a high risk of sarcopaenia, it is recommended by several international guidelines for community screening.^8^ However, its performance has been inconsistent across populations, often showing high specificity but low-to-moderate sensitivity, leading to concerns about under-detection if used alone for screening.^9^

## Methods

### Study design

This study adopted a cross-sectional design.

A total of11 public health clinics under the Ministry of Health Malaysia, spanning both urban and semi-urban settings in the states of Kedah, Perak, Terengganu, Negeri Sembilan, Kuala Lumpur, Johor and Sarawak, were involved in the study.

### Sample size

The sample size was calculated using the single-proportion formula: n=[DEFF×N×p(1-p)]/[d^2^/ Z^2^_1_-α/_2_×(N - 1) + p(1 - p)], where DEFF represents the design effect (1.5), p is the estimated prevalence of possible sarcopaenia (0.25), d is the margin of error or absolute precision (0.05) and Z is the Z-score corresponding to a 95% confidence level (1.96).^10^ The calculation was performed using OpenEpi version 3.01.^11^ Although the reported prevalence of sarcopaenia in Asian community- dwelling older adults typically ranges from 10% to 27%, we employed a conservative prevalence estimate of 50% to ensure maximum statistical power for estimating diagnostic performance. Based on an expected sensitivity and specificity of 80%, a desired precision of 5% and a 95% confidence level, the required sample size accounting for a 50% prevalence in the Malaysian older population and a 10% expected dropout rate was determined to be 547 participants.^12^ This calculation ensured that the study was sufficiently powered to estimate the diagnostic performance of the SARC-F score with the necessary clinical precision.

### Sampling method and participants

Patients aged >60 years attending government health clinics in Malaysia were recruited from J anuary to November 2024 using a convenience sampling approach. Patients residing in long-term care facilities and individuals who were cognitively impaired (Abbreviated Mental Test-4 scores of <3) were excluded from the study. Conversely, patients with hearing or visual impairments or literacy limitations who were accompanied by caregivers or family members were included. Caregivers acted as surrogate respondents or translators to ensure accurate and comprehensive data collection. Additionally, patients with pre-existing physical conditions that could affect clinical assessments, such as unilateral haemiparesis with muscle wasting, were excluded. These exclusion criteria were applied to ensure data quality and validity and accurately reflect participants’ health status.

### Data collection

Data were collected using a structured form, which consisted of three components:

1. Sociodemographic and clinical characteristics evaluation: Age, sex, ethnicity, comorbidities and polypharmacy (use of ≥5 medications) were recorded.
2. SARC-F questionnaire: The SARC-F questionnaire mainly captures strength-related difficulties in physical performance. Each component (strength, assistance in walking, rising from a chair, climbing stairs and falls) is scored from 0 to 2, yielding a total score of 0-10. A total score of ≥4 indicates a high risk for sarcopaenia.^7^ In this study, the English and Malay versions of the SARC-F questionnaire, previously validated for use among the Malaysian older population, were administered via face-to-face interviews,^13,14^ ensuring linguistic and conceptual accuracy.
3. HGS measurement: In this study, low muscle strength (HGS-defined) was used as a proxy for possible sarcopaenia due to feasibility. Other assessments (e.g. chair stand test and gait speed) were not included due to logistical constraints, including time, space and standardisation considerations across study sites. HGS yielded higher sensitivity and better overall diagnostic accuracy for possible sarcopaenia than gait speed or chair stand test result.^9^ HGS was measured using a calibrated Jamar hydraulic hand dynamometer (Model J00105, Lafayette Instrument Company, USA). All measurement procedures were performed by trained family medicine specialists.^4^ Participants were seated with elbows flexed at 90° and forearms in a neutral position. Two measurements were taken for each hand and averaged to ensure the reliability of grip strength data. The AWGS defines possible sarcopaenia as low muscle strength and/or reduced physical performance. Herein, low muscle strength was defined according to the AWGS 2019 sex-specific cut-off values: <28 kg for men and <18 kg for women.^4^

### Statistical analysis

Data were analysed using IBM SPSS Statistics Version 28.0 (IBM Corporation, Armonk, New York, United States). Normality of continuous data was assessed using the Shapiro–Wilk test. As HGS data were non-normally distributed, they were presented as medians and interquartile ranges (IQRs). The Mann–Whitney U test was utilised to compare the mean rank distributions of HGS between SARC-F risk groups (low risk vs high risk). The screening performance of the SARC-F score (score of ≥4) against the reference standard of HGS-defined possible sarcopaenia was evaluated by calculating sensitivity, specificity, positive predictive value (PPV), negative predictive value (NPV) and overall accuracy. The corresponding 95% confidence intervals (CIs) of sensitivity, specificity, PPV and NPV were calculated using Stata to meet standard reporting requirements. The agreement between the two tools was assessed using Cohen’s kappa coefficients, interpreted as follows: poor: <0.20, poor to fair: 0.21–0.40, moderate: 0.41–0.60, good: 0.61–0.80 and very good: 0.81–1.00.^15^ A P-value of <0.05 was considered statistically significant. Receiver operating characteristic (ROC) curve analysis was performed to evaluate the discriminatory performance of the SARC-F score in identifying possible sarcopaenia, using HGS as the reference standard. Possible sarcopaenia was defined based on low HGS according to the AWGS criteria and was treated as a binary state variable (presence vs absence of possible sarcopaenia). The total SARC-F score (ranging from 0 to 10), treated as a continuous test variable, was evaluated for its discriminative ability (i.e. its capacity to correctly classify individuals with and without possible sarcopaenia). The area under the ROC curve (AUC) and its corresponding 95% CI were calculated to quantify the overall diagnostic accuracy of the SARC-F score. The diagnostic accuracy of this score was interpreted based on the AUC as follows: no discriminative ability: 0.5, poor discrimination: 0.6–0.7, fair discrimination: 0.7–0.8, good discrimination: 0.8–0.9 and excellent discrimination: ≥0.9.^16^ This approach is standard practice in diagnostic validation studies; it allowed for the evaluation of the tool’s performance across all possible thresholds and confirmed whether the established cut-off of 4 was indeed the most appropriate for our specific study population.^7^

## Results

### Sociodemographic characteristics

A total of 578 eligible older adults participated in the study. The mean age was 71.3±6.1 years, and 54.7% (n=316) of the participants were women. The majority were Malay (54.8%), followed by those who were Chinese (28%) and Indian (8.3%). The most common comorbidities were hypertension (93.1%) and diabetes mellitus (62.3%). Polypharmacy was present in 63% of the participants (Table 1).

**Table 1:** Sociodemographic characteristics of the participants (N=578).

Sociodemographic variable	Frequency	Percentage
Age, mean (standard deviation)	71.3 (6.1)	
Age, year		
60-64	49	8.5
65-74	374	64.7
75-84	135	23.4
≥85	20	3.5
Sex		
Female	316	54.7
Male	262	45.3
Ethnicity		
Malay	317	54.8
Chinese	162	28.0
Indian	48	8.3
Others	51	8.8
Comorbid illness		
Hypertension	538	93.1
Diabetes mellitus	360	62.3
Heart diseases (ischaemic heart disease/heart failure)	94	16.3
Chronic kidney disease	93	16.1
Chronic lung disease	43	7.4
Stroke/transient ischaemic attack	35	6.1
Osteoporosis	15	2.6
Cancer	11	1.9
Dementia	5	0.9
Other chronic diseases	28	4.8
Number of prescribed medications		
<5	214	37.0
≥5	364	63.0
Possible sarcopaenia		
No	281	48.6
Yes	297	51.4

**Table 2:** Comparison of HGS according to the SARC-F risk category and sex.

SARC-F category	HGS in men (kg)			HGS in women (kg)		
	n	Median (IQR)	P-value^*^	n	Median (IQR)	P-value^*^
High risk SARC-F score of ≥4 Low risk SARC-F score of 0–3	45 217	19.0 (12.0) 28.0 (14.3)	<0.001	93 223	14.0 (8.3) 18.0 (8.0)	<0.001

* Mann–Whitney U test. Low muscle strength according to the Asian Working Group for Sarcopenia 2019 sex-specific cut-off values: HGS of <28 kg for men and <18 kg for women. HGS: hand grip strength, IQR: interquartile range.

**Table 3:** Diagnostic accuracy of the SARC-F score (≥4) against hand grip strength for predicting possible sarcopaenia (N=578).

SARC-F category	Hand grip strength		Kappa value	P-value	Sn (95% CI)	sp (95% CI)	PPV (95% CI)	NPV (95% CI)	Accuracy (%)
	Low n (%)	Normal n (%)							
High risk SARC-F score of ≥4	103 (74.6)	35 (25.7)	0.219	<0.001	34.7 (30.8-38.6)	87.5 (84.9-90.2)	74.6 (71.1-78.2)	55.9 (51.9-60.0)	60.4
Low risk SARC-F score of 0–3	194 (44.1)	246 (55.9)							

Sn: sensitivity, Sp: specificity, PPV: positive predictive value, NPV: negative predictive value, CI: confidence interval.

### Prevalence of possible sarcopaenia and distribution of grip strength

Based on HGS, the prevalence of possible sarcopaenia was 51.4% (n=297) (Table 1). The median HGS for the non-sarcopaenia and possible sarcopaenia groups was 28.0 kg (IQR=13.5) and 15.0 kg (IQR=7.0), respectively.

### Association between the SARC-F score and HGS

The participants were classified into high-risk (SARC-F score of ≥4) and low-risk (SARC-F score of 0–3) groups based on the SARC-F screening results. HGS was used as the reference standard for identifying possible sarcopaenia according to the AWGS 2019 criteria.

There was a significant association found between the SARC-F risk category and HGS in both men and women. Among the male participants, those classified as having a high risk based on the SARC-F score demonstrated substantially lower HGS than those classified as having a low risk (19.0 kg [IQR=12.0] vs 28.0 kg [IQR=14.3]; P<0.001). Similarly, among the female participants, the high-risk SARC-F group had significantly lower HGS than the low-risk SARC-F group (14.0 kg [IQR=8.3] vs 18.0 kg [IQR=8.0]; P<0.001).

The findings indicated that higher SARC-F scores were consistently associated with reduced muscle strength across both sexes, supporting the construct validity of the SARC-F score in reflecting functional muscle impairment (Table 2).

### Discriminatory performance of the SARC-F score in identifying possible sarcopaenia

**Figure 1:**
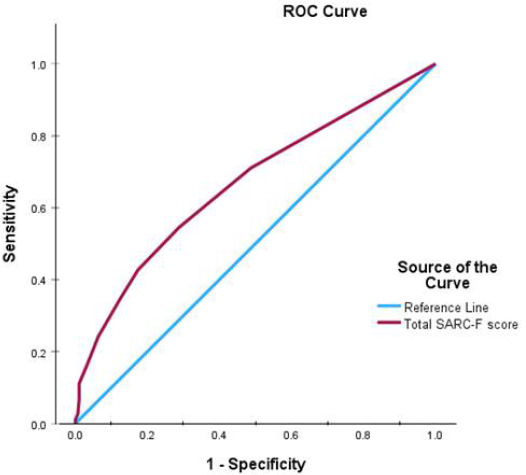
ROC curve illustrating the screening performance of the total SARC-F score in identifying possible sarcopaenia, using hanf grip ftrength as thfreference scandard. The diagonal line repreeents the lcne oCno discrimination (AUC=0.50). The SARC-C score demonstrated an AUC of 0.666 (95% CI=0.622–0.710), indicnting poor discriminarive ability. ROC: receiver operating charaeoerietic, AUC: area unCer rhe ROC curve, CI: confidence intervcl.

The AUC for the total SARC-F score in detecting possible sarcopaenia, using HGS as the reference standard, was 0.67 (95% CI=0.622–0.710; P<0.001), which indicated poor discriminative ability (Figure 1).^14^ Although the SARC-F score demonstrated significant discrimination compared with chance, its overall accuracy in distinguishing older adults with and without possible sarcopaenia was limited.

### Diagnostic accuracy of the SARC-F score for identifying possible sarcopaenia

With HGS as the objective reference, the SARC-F score demonstrated a sensitivity of 34.7% (95% CI=30.8–38.6) and a specificity of 87.5% (95% CI=84.9–90.2) (Table 3). The PPV was 74.6% (95% CI=71.1–78.2), indicating that approximately three-quarters of individuals classified as having a high risk based on the SARC-F score had low HGS. In contrast, the NPV was 55.9% (95% CI=51.9–60.0), suggesting that nearly half of those classified as having a low risk based on the SARC-F score still met the criteria for possible sarcopaenia.

The overall diagnostic accuracy of the SARC-F score was 60.4%. The agreement between the SARC-F score- and HGS-based classifications was poor to fair, with a Cohen’s kappa coefficient of 0.219, which was significant (P<0.001).

### Distribution of HGS according to the SARC-F risk category

Fijeire2 shows thtk the participants in the high-riskgroup (SARC-F score of ≥4) had lower median HGS and a generally narrower distribution than those in the low-risk group (SARC-F srore of 0–3). The entire distribution of HGS in the high-risk group was shifted towards lower values, indicating consistently reduced muscle strength, whereas the low-risk group demonstrated higher median values, greater variability and the presence of higher-value outliers.

**Figure 2:**
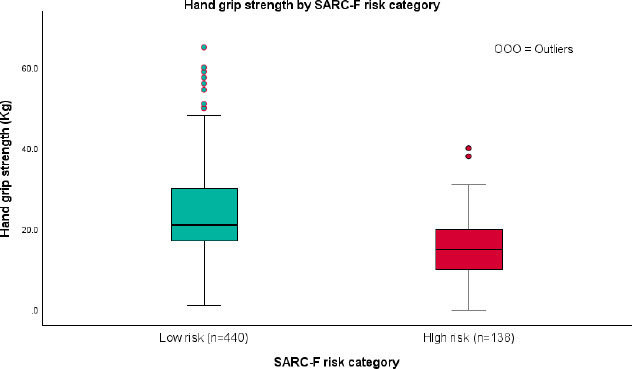
Boxplot illustrating a clear inverse relationship between hand grip strength (kg) and the SARC-F risk category. The box represents the interquartile range; the line inside the box indicates the median;and individta^-^ dote represent the observed values. (Outliers, defined os value-exceeding S.5 timet the interqrartile tange, are indicated by epen circles. Thir uisorl -rttern supports a potential inverse as-ociation betwren the SARC-F score and muscle sirengtd.

## Discussion

About one in two older adults attending health clinics had possible sarcopaenia in our study, which is higher than typically reported in community-based populations. The use of screening criteria aligned with the AWGS, focusing on muscle strength based on HGS, may increase case detection compared with the assessment of physical performance alone.^4^ Additionally, the sampling was conducted by family medicine specialists handling patients with a higher burden of comorbidities or older higher-risk groups, which have been associated with increased prevalence of possible sarcopaenia.

This study evaluated the performance of the SARC-F questionnaire against objective HGS measurement for detecting possible sarcopaenia in a Malaysian primary care setting. The key finding is that while a positive SARC-F score of >4 was strongly indicative of low muscle strength (high specificity and PPV), the tool missed a substantial proportion of cases (low sensitivity), demonstrating only fair agreement with the reference standard.

The inverse association between the SARC-F score and median HGS, significant for both sexes, is consistent with the construct of the tool and findings from other populations.^17^ This confirms that the subjective deficits captured by the SARC-F score are related to measurable physiological decline. The high specificity (87.5%) and PPV (74.6%) indicate that when the SARC-F score reflects a positive result, it is likely that an individual has low muscle strength. This makes it a useful ‘rule-in’ tool in clinical practice, where a positive screen can confidently prompt further assessment or intervention.

However, the low sensitivity (34.7%) is a major limitation, similar to reports from other Asian studies where sensitivities ranged from 20% to 46%.^9,18^ This indicated that 65.3% of the older adults with objectively defined possible sarcopaenia were missed by the SARC-F screening. This high false-negative rate suggests that many older adults may not perceive or report functional limitations until muscle weakness is severe or that they may adapt their lifestyles to avoid challenging tasks, possibly causing further deterioration in sarcopaenia. Relying solely on the SARC-F score for screening would therefore fail to identify a large pool of at-risk individuals who could benefit from early intervention.

Given the overall accuracy of 60.4% and the modest NPV of 55.9%, a negative SARC-F result does not reliably exclude possible sarcopaenia. The poor-to-fair kappa agreement (0.219) indicates limited agreement and reinforces that the SARC-F score and HGS are not interchangeable measures. In the context of a busy health clinic, these findings suggest a pragmatic two-step screening approach. The SARC-F questionnaire, which can be rapidly completed and does not require equipment, can serve as an initial filter. For those screening positive (score of ≥4), the diagnosis of possible sarcopaenia is highly probable, and management plans can be initiated. For those screening negative (score of <4), given the high rate of missed cases, we suggest a modification in the AWGS criteria that a more proactive strategy with HGS measurement is needed. In health clinics where resources permit, routine annual HGS measurement for all older adults as part of a comprehensive geriatric assessment would be ideal. Where this is not feasible, targeted HGS testing for high-risk subgroups such as those with multimorbidity including heart failure, diabetes mellitus, chronic kidney disease, chronic obstructive pulmonary disease, geriatric syndromes such as recurrent falls and unexplained weight loss despite a negative SARC-F result could improve case detection.^6,19-21^

In this study, the SARC-F score demonstrated a significant but limited ability to discriminate possible sarcopaenia with HGS as the reference standard, with an AUC of 0.67, indicating poor discriminatory performance. This finding is consistent with existing evidence suggesting that while the SARC-F questionnaire is a simple and practical screening tool, it may lack sufficient sensitivity for accurately identifying early or probable sarcopaenia in community-dwelling older adults. Nevertheless, the significant discrimination suggests that the SARC-F questionnaire retains some clinical utility, particularly as an initial screening instrument in primary care and community settings where rapid assessment is required. These findings support current recommendations that the SARC-F score should be complemented with objective strength measures to improve the detection of possible sarcopaenia.^6^

This study has limitations in confirming possible sarcopaenia. The diagnosis of possible sarcopaenia was based solely on HGS without assessing physical performance due to logistic limitations. Individuals with normal strength but impaired function may have been missed, which could affect the evaluation of the screening performance of the SARC-F score. For the complete construct of possible sarcopaenia, further studies should include both low muscle strength and physical performance. Another potential limitation of this study is that factors such as age, sex, comorbidities and body mass index were not analysed, yet they may influence both the SARC-F score and HGS. Future studies should adjust for potential confounders to better assess the independent predictive value of the SARC-F score.

## Conclusion

The SARC-F questionnaire is specifically used for identifying possible sarcopaenia but lacks sensitivity. Its use as a standalone universal screening instrument in health clinics may lead to significant underdiagnosis. A combination of subjective screening (SARC-F questionnaire) followed by objective confirmation (HGS measurement) for targeted groups offers an effective strategy for improving the early detection of possible sarcopaenia in resource-conscious settings including Malaysian public health clinics. Future research should explore the feasibility and barriers of such a combined approach among primary healthcare providers and patients.

## Data sharing statement

Data are available upon request from the corresponding author.

**How does this paper make a difference in general practice?**

- This research indicated that combining the SARC-F questionnaire with hand grip strength assessment may improve case finding for possible sarcopaenia in health clinics compared with using the SARC-F questionnaire alone for screening as currently recommended by the Asian Working Group for Sarcopenia.
- The study supports the enhancement of handgrip dynamometer availability in health clinics for better detection of possible sarcopaenia, especially among high-risk older adults, despite having negative SARC-F screening results.
- Future research evaluating both low muscle strength and physical performance should be conducted for better evaluation of the screening performance of the SARC-F score.
